# Assembly of Multilevel Nanoconstructs with Negatively Charged Lipid Envelope and Features of Its Interaction with Protein Corona

**DOI:** 10.3390/nano16120743

**Published:** 2026-06-14

**Authors:** Ilya S. Dovydenko, Anna V. Epanchintseva, Julia E. Poletaeva, Elena I. Ryabchikova

**Affiliations:** Institute of Chemical Biology and Fundamental Medicine, Siberian Branch of Russian Academy of Science, Lavrent’ev Avenue. 8, 630090 Novosibirsk, Russia; dovydenko_il@1bio.ru (I.S.D.); annaepanch@niboch.nsc.ru (A.V.E.); poletaeva@niboch.nsc.ru (J.E.P.)

**Keywords:** gold nanoparticles, enveloping in negatively charged phospholipids, TEM and DLS characteristics, protein corona interaction with lipid envelope

## Abstract

Despite extensive research, formation and properties of protein corona (PC) remain largely unknown. The composition and properties of PC are unique to each particle type. Our research focuses on multilevel nanoconstructs (MLNCs) containing a core (AuNP coated with oligonucleotide) encapsulated in lipid envelope (LE). We are developing particles of this type as nucleic acid delivery systems and platforms for studying PC on lipid surfaces. The goal of this work is to optimize the assembly of MLNCs with a negatively charged LE encapsulating a negatively charged core. Magnesium ions successfully acted as electrostatic bridges between like-charged components to facilitate self-assembly. The resulting particles were characterized using DLS (hydrodynamic diameter of ~36 nm) and TEM, which revealed stable LE. However, we encountered a critical issue: mechanical strength of the phosphatidylcholine/phosphatidic acid/cholesterol envelope proved to be highly sensitive to centrifugation forces and interactions with proteins. Incubation with albumin destabilized the LE, resulting in core release. In contrast, exposure to serum maintained the integrity of LE, allowing isolation of MLNC particles bearing PC. These results demonstrate that the assembly protocol can be adapted to negatively charged lipid compositions. However, stability of MLNCs during isolation is strictly dependent on medium protein composition. Thus, MLNCs represent a valuable platform for studying the interactions of LE with the PC.

## 1. Introduction

Modern nanopharmaceutical design tools are extremely diverse, and lipid-based constructs occupy an important, if not central, place among them. The enormous diversity of natural lipid molecules allows for the assembly of a drug framework with desired properties, and the good biocompatibility of lipids facilitates overall drug formulation [1,2,3,4]. Lipid constructs are present on the market as delivery systems for vaccines and drugs, including transporters of toxic compounds for cancer treatment [5,6,7,8,9]. Despite the undeniable success of lipid constructs in pharmaceutical development, unsolved problems remain in this field, one of which is the development of tools for “controlling” nanoconstructs using a protein corona [10,11]. The formation of a protein corona on any nanoparticle (NP) entering any biological fluid is an indisputable fact. The fundamental principles of this process have been established: the corona consist of two layers: “hard corona” (a protein layer of constant composition, firmly bound to the NP surface) and a “soft corona,” of variable composition, the proteins of which are mobile and easily replaced by others [12,13,14]. It is the soft corona proteins that determine the uniqueness of the protein corona on each nanoparticle, which depends on the molecular composition of the biological fluid capturing the NP, its physical parameters (temperature, flow rate, etc.), as well as the composition and surface shape of the particles themselves [15]. Another established fundamental fact is the crucial role of the soft protein corona in determining the nature of particle interactions with body cells, including drug target cells [16,17,18,19,20]. The properties of NPs can, to varying degrees, be replaced by the properties of the soft protein corona, necessitating the study of its properties and molecular composition.

**Figure 1:**
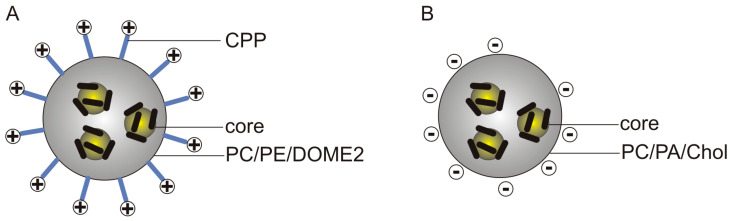
Schematic diagrams of multilayered nanoconstructs. (**A**)—MLNC-1, (**B**)—MLNC-2.

Currently, there are several approaches to studying the composition of the full protein corona and its component, the soft corona [21]. One such approach is the immobilization of proteins on the surface of NPs using click chemistry [20], and the photoactivation method proposed by our team [22]. We implemented our approach using multilayer nanoconstructs (MLNC-1) with a lipid envelope capable of delivering siRNA into cells (Figure 1A) [23]. The MLNC-1 core is a non-covalent complex of oligonucleotides with spherical gold nanoparticles (AuNPs), hereafter referred to as the “core.” In the “complete” construct, several cores are “encapsulated” within a lipid envelope (Figure 1A).

The lipid layer of MLNC-1 is composed of egg phosphatidylcholine (PC)/1,2-dioleoyl-sn-glycero-3-phosphoethanolamine (DOPE)/2-[[4-dodecylamino-6-oleylamino-1,3,5-triazine-2yl]-(2-hydroxyethyl)amino]ethanol (DOME2) in a molar ratio of 4.5/4.5/1. The DOME2 lipid is an ionizable cationic lipid that served as a link between the lipid envelope and the core during their assembly. Under acidic conditions, DOME2 is protonated and carries a positive charge, which ensures the sorption of negatively charged cores of the lipid film and their enveloping into a lipid particle. The surface of MLNC-1 is modified with a cell-penetrating protein (CPP). The process for MLNC-1 obtaining is described in detail in [23].

MLNC-1 has proven to be a promising experimental object. In addition to efficient siRNA delivery into cells [23], its lipid surface was able to photofix the full protein corona, allowing us to determine the protein composition of the soft corona [24]. The presence of several AuNPs within the nanoconstruct (Figure 1A) increased the density of MLNC-1 particles and allowed using low-speed centrifugation for their efficient separation from non-enveloped cores and empty lipid particles. Using this property, we were able to easily separate MLNC-1 bearing a hard protein corona or a photo-fixed full protein corona from unbound components of the biological environment and determine the protein composition of the coronas [24]. In addition, electron-dense AuNPs serve as a clear marker of MLNC-1, allowing visualization of nanoconstructs in a transmission electron microscope (TEM) and monitoring the integrity of the structures during experiments, as well as studying the interaction of MLNC1 with the cell [25].

Our studies have shown that MLNC-type particles assembled in the sequence: AuNPs–non-covalently bound RNA layer–lipid layer (envelope) can potentially be used to examine the relationship between the composition of the lipid layer and the composition of the protein corona formed on the surface of this layer. The MLNC construct also provides possibility to study the mechanisms of MLNC penetration into cells and their intracellular transport. However, the proposed approach to MLNC-1 assembly has a limitation: it was designed for lipid envelopes containing ionizable cationic lipids [26].

The objective of this study is to modify our MLNC-1 assembly protocol [26] for the assembly of multilayer nanoconstruct containing negatively charged phospholipids. In fact, we aimed to create a new type of MLNC with both a negatively charged core and lipid surface (MLNC-2) (Figure 1B), and to study the interaction between the MLNC-2 lipid envelope and the proteins that form the corona on the surface of the new particle. The creation of a nanoconstruct containing a negatively charged core and a negatively charged lipid envelope will expand the range of tools for studying the properties of the protein corona.

## 2. Materials and Methods

### 2.1. Chemicals

Tetrachloroauric acid trihydrate (HAuCl_4_·3H_2_O) was purchased from Aurat (Moscow, Russia), and DNA phosphoramidites for oligonucleotide synthesis were acquired from Sigma-Aldrich (Hamburg, Germany). Sodium chloride (NaCl) and magnesium sulfate (MgSO_4_) were bought from Honeywell (Seelze, Germany), sodium citrate dihydrate (Na_3_C_6_H_5_O_7_·2H_2_O) from Fluka (Buchs, Switzerland) and uranyl acetate from SPI (West Chester, PA, USA). Egg phosphatidylcholine and phosphatidic acid were acquired from Avanti (Alabaster, AL, USA), whereas cholesterol was obtained from Sigma-Aldrich. Sucrose (C_12_H_22_O_11_) was bought from Panreac (Barcelona, Spain) as well as solvents for oligonucleotide synthesis. Trichloromethane (chloroform, CHCl_3_) and methanol (CH_3_OH) were purchased from Reachem (Moscow, Russia). Water was purified by means of a Simplicity 185 water purification system (Millipore, Burlington, MA, USA) and had a resistivity of 18.2 MΩ·cm at 25 °C.

### 2.2. Oligonucletide Synthesis

Fluorescein labeled T_26_ oligodeoxyribonucleotid for core preparation was synthesized on an automatic ASM-800 DNA/RNA synthesizer (Biosset, Novosibirsk, Russia) at a 0.4-µmol scale using 2′-deoxyphosphoramidites and solid-phase phosphoramidite synthesis protocols [27], optimized for the synthesizer. The oligonucleotide was cleaved from the solid support, and the protective groups were removed from the nucleobases by treatment with 30% ammonia hydroxide for 16 h at 25 °C. The crude oligonucleotides were then precipitated with ethanol in the presence of 0.3 M NaCl. The resulting precipitate was washed with ethanol, dried, and dissolved in 100 µL of deionized water. After, oligonucleotide were purified by PAGE.

### 2.3. Preparation of Core Nanoparticles

AuNPs were prepared using the classic citrate reduction procedure [28]. The size of AuNPs and their dispersity were determined by TEM and DLS. The concentration of AuNPs was calculated from absorbance at 520 nm using an extinction coefficient of (8.78 ± 0.06) × 10^8^ M^−1^ cm^−1^ [29].

For gold cores, a solution containing 3.6 nM citrate-stabilized AuNPs, 0.7 μM of T_26_-FAM, 5.6 mM NaCl (Honeywell, Seelze, Germany), and 0.1 mM MgSO_4_ (Honeywell, Seelze, Germany) in a total volume of 3.47 mL was incubated at 25 °C and shaked at 800 rpm overnight. This reaction mixture contains 12.5 pmol of AuNPs. After that, cores were sedimented at 16,100× *g* and 25 °C for 30 min and supernatants were discarded.

### 2.4. Preparation of a Lipid Film

The lipid film was prepared in a clean glass 50 mL round-bottom flask: 350 μL of 1 mM egg phosphatidylcholine and 100 μL egg phosphatidic acid in a CHCl_3_/CH_3_OH mixture (1:1) and 50 μL of 1 mM cholesterol in CHCl_3_ were added to 5 mL of CHCl_3_. Solution of lipids was well mixed in an ultrasonic bath. The solvent was then evaporated at a temperature of 25 °C and a pressure of 14 mmHg. The resultant lipid film was then dried in vacuum in a desiccator for 2 h to remove traces of the organic solvents. All the procedures for obtaining the lipid film were carried out in an argon atmosphere.

### 2.5. Assembly of the MLNC-2

MLNC-2 assembly was performed similarly to [26] with minor adjustments to the reaction parameters, as described below. The MLNC-2 particles were obtained via a two-step process. The core pellet was diluted with 0.08 mM freshly prepared magnesium sulfate solution to a final volume of 5 mL; the resulting solution was added to the flask containing the thin lipid film and hydrated at 25 °C with constant rotation for 10 min. The flask was then placed on ice to cool the contents and then sonicated for 10 min at 90 W in an ultrasonic bath filled with floating ice. To prepare a 0.08 mM magnesium sulfate solution, argon-purged water was used. The described technique allows for obtaining particles of a fairly reproducible size (see Figure S1).

### 2.6. Modification of FBS with PACL

A 50% fetal bovine serum (FBS) (Thermo Fisher Scientific, Waltham, MA, USA) solution in 1 mM phosphate buffer (pH 7.2) was mixed with 12 µL of 0.5 M PACL in DMSO and incubated at 20 °C under constant stirring for two hours. Excess PACL was removed using Bio-Spin 6 gel filtration columns (Bio-Rad, Hercules, CA, USA) according to the manufacturer’s protocol. Due to the light sensitivity of PACL, all procedures with this compound were carried out in a dark room illuminated by a red lamp. PACL (4-azido-N-[3-[3-(2,5-dioxopyrrol-1-yl)propanoylamino]propyl]-2-nitro- benzamide) was synthesized in the laboratory of organic synthesis of ICBFM SB RAS (Novosibirsk, Russia).

### 2.7. Incubation of the MLNC-2 with Human Serum Albumin or with Native or Modified Serum

The protein corona on MLNC-2 particles was formed similarly to [24]. To 1 mL of MLNC-2, 250 µL of a human serum albumin (HSA) solution (20.5 mg/mL; Sigma-Aldrich, Hamburg, Germany), or a 50% FBS solution in 1 mM phosphate buffer (pH 7.2), or a PACL-modified FBS solution (see Section 2.6) was added. The resulting mixture was gently mixed by pipetting and incubated for 20 min at 25 °C. When working with PACL-modified FBS, all procedures were performed in a dark room.

For cross-linking of PACL-modified FBS proteins with the MLNC-2 surface, the mixture of MLNC-2 with PACL-modified FBS was exposed to UV irradiation at a wavelength of 310 nm and a power of 8.2 mW/cm^2^ for 1 min.

### 2.8. Purification of the MLNC-2 or MLNC-2-Bearing Protein Corona

MLNC-2 or MLNC-2 with protein corona were purified by centrifugation in an aqueous 7% sucrose solution (ρ = 1.0279 g/mL) at 300× *g* and 5 °C for 140 min. Bright red-colored fractions containing the resulting MLNC-2 were collected into separate 1.5 mL tubes and concentrated by centrifugation at 300× *g* and 5° C for 15 min.

The particles bearing protein corona were washed tree times with 7% sucrose solution; particles were sedimented at 300× *g* and 5° C for 15 min

### 2.9. Gel Electrophoresis

Proteins bound to the surface of MLNC-2 or to the core surface were analyzed by sodium dodecyl sulfate (Sigma-Aldrich, Hamburg, Germany) polyacrylamide gel electrophoresis (SDS-PAGE) using a 7% polyacrylamide gel (Dia-M, Moscow, Russia) under Laemmli conditions, followed by staining with Coomassie Brilliant Blue (Dia-M, Moscow, Russia). The entire material obtained after isolation and washing (see Section 2.8) was loaded into the gel pocket.

### 2.10. Examination of the Structure and Quality of the MLNC-2 Samples

#### 2.10.1. DLS

Suspensions of core and MLNC-2 nanoparticles in 7% sucrose were analyzed by dynamic light scattering (DLS) using a Malvern Zetasizer Nano instrument (Malvern Instruments, Worcestershire, UK) according to the manufacturer’s instructions. This method enabled characterization of the particle suspensions by the following parameters: hydrodynamic diameter and polydispersity index (PDI). Each sample was measured at least ten times. Results are presented as mean ± SD.

#### 2.10.2. TEM

All samples were examined in TEM, similarly to the method described in [26]. Briefly, 5 μL of each sample was placed on a formvar-coated copper 200-mesh grid. After adsorption for 1 min, liquid was removed with a pipette and the grid was applied on a drop of 0.5% aqueous uranyl acetate solution (EMS, Hatfield, PA, USA) for 10 s, followed by drying the grid with filter paper. A JEM-1400 transmission electron microscope Jeol (Tokyo, Japan) equipped with a Veleta digital camera EM SIS (Münster, Germany) was used for imaging. For each sample, at least five meshes from four different areas of the grid were examined. Particle sizes were determined using iTEM software, version 5.2 EM SIS.

## 3. Results and Discussion

### 3.1. Justification for the Selection of Components for Obtaining MLNC-2

In this work, we applied previously developed approaches to the assembly of multilevel nanoconstruct on AuNPs [26] to obtain a similar nanoconstruct, the envelope of which is formed by negatively charged lipids. During the course of our research, we discovered a number of features of the new particles, which is information we would like to share with other researchers working in this field in the hope that it will be useful.

In our previous studies [26], particles of the MLNC-1 type (Figure 1A) were obtained in a self-assembly process based on the electrostatic interaction of negatively charged cores (a non-covalent complex of siRNA with AuNPs) with a lipid film carrying a positive charge under acidic conditions. Due to the heavy core of MLNC-1, these particles can be easily separated from non-enveloped cores and empty lipid particles by low-speed centrifugation on sucrose or glycerol cushions [26]. The same feature of MLNC-1 was successfully used in the study of the protein corona formed on their lipid surface [24].

The aim of this work is to prepare a multilevel nanoconstruct with a negatively charged lipid coating and to study the interaction of the lipid envelope with the protein corona, as was previously done with MLNC-1 particles [24].

To assemble MLNC-2 particles (Figure 1B), magnesium ions were chosen as the binding component between the negatively charged cores and the negatively charged lipid envelope. This choice was based on our previous experience. Thus, the cores maintained colloidal stability in an acidic phosphate buffer (pH 4.5), which, when a suspension of cores was added to the lipid film in the time of assembly of MLNC-1, ensured the protonation of DOME2 [26]. In this study, we found that phosphate anions compete with the phosphate groups of the nucleic acid located on the surface of the cores, and thereby negatively affected the efficiency of core encapsulation in the lipid envelope. The addition of divalent magnesium ions increased encapsulation efficiency without replacing the buffer system that maintains the colloidal stability of the cores. This led us to believe that magnesium ions could act as a binding component between the cores and the lipid envelope with negatively charged phospholipids. Egg phosphatidyl choline (PC, a zwitterionic phospholipid), egg phosphatidylic acid (PA, a negatively charged phospholipid), and cholesterol (a neutrally charged lipid) were used for the lipid envelope of MLNC-2.

At the first stage of the experimental work, we selected the composition of the lipid mixture and determined the allowable concentration of magnesium. We did not limit ourselves to using only zwitterionic phospholipids and neutral lipids, since in the absence of charged groups on the surface of the final particles, the surface potential will be around zero mV. Such a potential, during further manipulation, in particular, purification by centrifugation, will inevitably lead to the particles sticking together and merging, which will significantly distort the experimental data

### 3.2. Choice of Lipid Composition

The following lipids were used to build the lipid envelope around the cores: egg PC, egg PA and cholesterol. We varied the ratio of PA and PC in the lipid mixture, aiming for |ζ| > 25 mV on the particle surface; cholesterol fraction was constant at 10 mol%. The amount of PA in the lipid mixtures corresponded to 10, 20, 40 and 50 mol% (Table 1).

For this experiment, thin lipid films of four compositions (Table 1) were prepared, hydrated for 15 min in a 0.9% NaCl solution, and then sonicated. The ζ-potential was determined for the obtained particles (Table 1): 10 mol% PA in the lipid mixture results in |ζ| < 25 mV, while increasing the proportion of PA to 20 mol% and above provides the required |ζ| > 25 mV. For further experiments, a mixture of PC/PA/Cholesterol 70/20/10 mol% was chosen.

### 3.3. Selection of Magnesium Ion Concentration for Optimal Assembly of MLNC-2

Because the colloidal stability of cores depends on both the ionic strength and the type of ions present in the solution, we preliminarily assessed the stability of cores in a magnesium sulfate solution. For cores, it was decided to use a simpler and cheaper option: the siRNA used in MLNC-1 was replaced with a 26-mer oligodeoxythymidylate with fluorescein (T_26_- FAM) attached to the 5′ end. A fluorophore-labeled oligonucleotide was used because the adsorption of fluorophore-labeled oligonucleotide on the surface of AuNPs has been shown previously [30,31].

**Figure 2:**
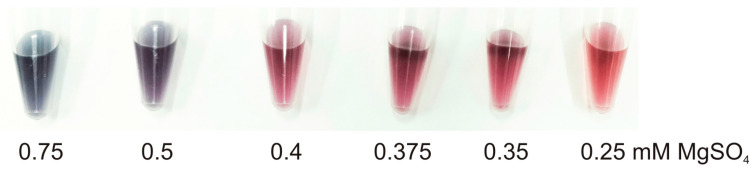
Colloidal stability of cores in the presence of magnesium ions. The image shows the change in the core suspension color caused by aggregation of cores in magnesium sulfate solution.

The sediment containing the concentrated cores was resuspended in a magnesium sulfate solution. Depending on the magnesium sulfate concentration, the change in suspension color was observed (Figure 2).

Mixing the core sediment with a 0.75 mM magnesium sulfate solution resulted in a change in the core suspension color from red to blue-gray; the particles settled within half an hour. Similar changes were observed in a solution with a magnesium ion concentration of 0.5 mM. Signs of colloidal stability of the cores were observed with a magnesium sulfate concentration of 0.4 mM and below, so we used magnesium sulfate at a concentration of ≤0.4 mM to assemble the new multilevel nanoconstruct, MLNC-2.

### 3.4. MLNC-2 Assembly Optimization

**Figure 3:**
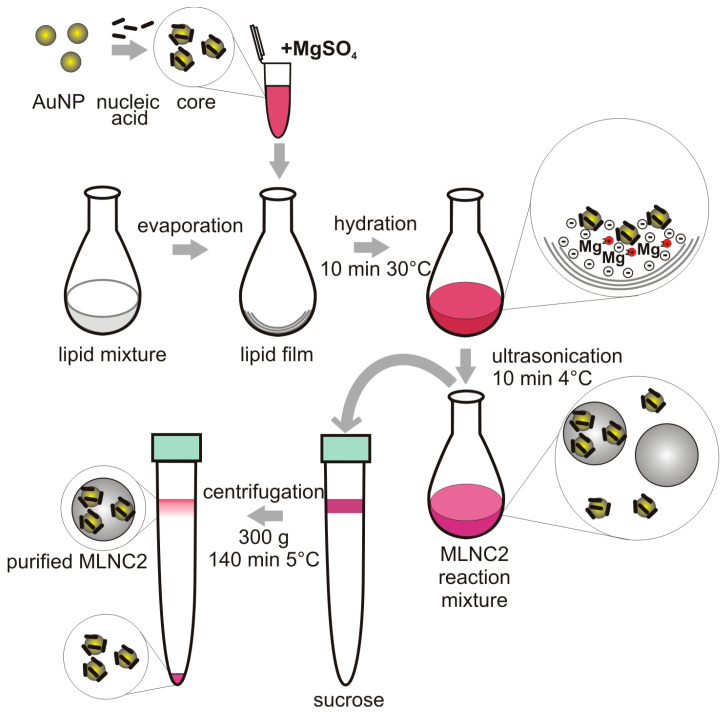
Assembly flowchart of the MLNC-2 nanoconstruct. Upper line: formation of cores from AuNPs and nucleic acid, and the preparation of a thin lipid film; hydration of a core suspension in the presence of magnesium ions. Bottom line: sonication of the resulting mixture, purification of MLNC-2 in sucrose solution by low-speed centrifugation.

The scheme of MLNC-2 assembly is shown in Figure 3.

To prepare a lipid film for MLNC-2 assembly, lipid solutions with a concentration of 1 mM were mixed in a clean round-bottomed flask (50 mL): cholesterol (50 µL), PC (350 µL) and PA (100 µL). The resulting mixture was diluted with 5 mL of chloroform and mixed well by ultrasonication for 30 s. The lipid mixture was then slowly evaporated on a rotary evaporator to form a thin film on the inner surface of the flask (Figure 3). The pressure at the time of evaporation was 14 mm Hg, the rate of evaporation of organic solvents was regulated by the bath temperature (from 25 °C) and also by the volume of chloroform in the flask receiver of the rotary evaporator (50 mL was poured). The 5.5 mL solvents evaporated in approximately 15 min. Next, the flask with the formed film was dried in a desiccator under reduced pressure for two hours. The cores were then encapsulated in a lipid envelope by hydrating the thin lipid film with a suspension of cores with magnesium sulfate (Figure 3). After hydration, ultrasonic treatment was performed. It is important that the mixture be pre-cooled to 4 °C and that the ultrasonic treatment (10 min) be performed strictly at this temperature (Figure 3). Otherwise, the MLNCs will be destroyed.

**Figure 4:**
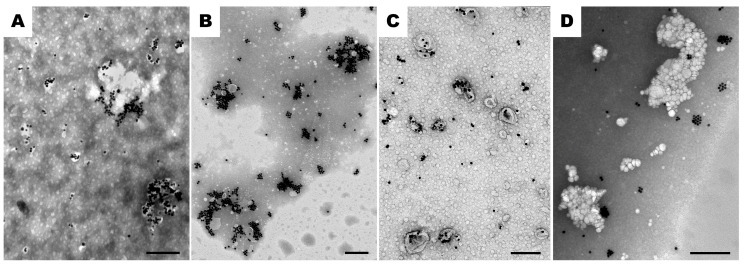
Representative images of the MLNC-2, obtained at different magnesium ions concentration, and sonicated. The images (**A**–**D**) correspond to 0.12, 0.1, 0.08-, and 0.06 mM concentrations. TEM. Negative staining with 0.5% uranyl acetate. Length of scale bars corresponds to 200 nm.

As shown in Figure 2, the cores maintain colloidal stability in the range of magnesium ion concentrations from 0.25 to 0.4 mM. However, using magnesium sulfate concentrations in this range did not provide consistently reproducible assembly; therefore, we decided to reduce the magnesium ion concentration to 0.12, 0.1, 0.08, and 0.06 mM, Figure 4 shows TEM images of the samples. The best result was obtained at a Mg^2+^ concentration of 0.08 mM. The resulting suspension contained MLNC-2, empty lipid particles, and non-enveloped cores (Figure 3 and Figure 4).

### 3.5. Purification of MLNC-2

The next step was to optimize the purification conditions for the MLNC-2 suspension, removing empty lipid particles and non-enveloped cores (Figure 3), using centrifugation. The particle suspension was layered on a 58% sucrose cushion, and then separated at 2000× *g* for 15 min at 25 °C [26]. Under these conditions, MLNC-2 did not penetrate the cushion; the sample concentrated at the density’s boundary of the two solutions. The cores of a number of particles have gone beyond the envelope, and, accordingly, the yield of MLNC-2 has decreased.

We initially concluded that the lack of separation in 58% sucrose solution was due to the significantly lower density of MLNC-2 compared to the density of the sucrose cushion itself, which is why the sucrose concentration was reduced. However, we observed a similar pattern when the sucrose concentration was reduced to 7%: the particles concentrated at the density boundary, and non-enveloped cores accumulated at the bottom of the test tube after centrifugation. These observations suggest that the density of MLNC-2 particles is less than the density of a 7% sucrose solution. To verify this, an additional experiment was conducted to evaluate the buoyancy of MLNC-2 particles in sucrose solutions from zero to 9%. For this purpose, 1 mL of MLNC-2 suspension in 10% sucrose was placed on the bottom of a 2 mL test tube, and then 1 mL of sucrose with a concentration of 0–9% was layered on this bottom layer. The tubes were centrifuged at 5 °C and 300 g for 30 min. Inspection of the tubes after centrifugation revealed no diffusion of MLNC-2 into the more dilute sucrose layer. The experiment was repeated without additional acceleration, and the tubes were incubated at 5 °C in a refrigerator for 16 h. The result: MLNC-2 particles did not float, indicating that their density is greater than that of the 9% sucrose solution. Therefore, the particle density was not the reason for the lack of MLNC-2 separation on the 7% sucrose pad.

Another reason for the lack of separation on a 7% sucrose cushion may be the destruction of MLNC-2 during centrifugation. Apparently, the mechanical strength reserve of the lipid envelope of MLNC-2, consisting of PC/PA/Chol (7:2:1), is much less than that of the lipid envelope of MLNC-1, which was represented by PC/PE/DOME2 (4.5:4.5:1). Thus, the reduction in hydrodynamic resistance due to the decrease in the viscosity of the medium during the transition from 58% sucrose to 7% sucrose is insufficient, and therefore, in the presence of centrifuge acceleration, the core breaks through the lipid envelope, and leaves MLNC-2.

**Figure 5:**
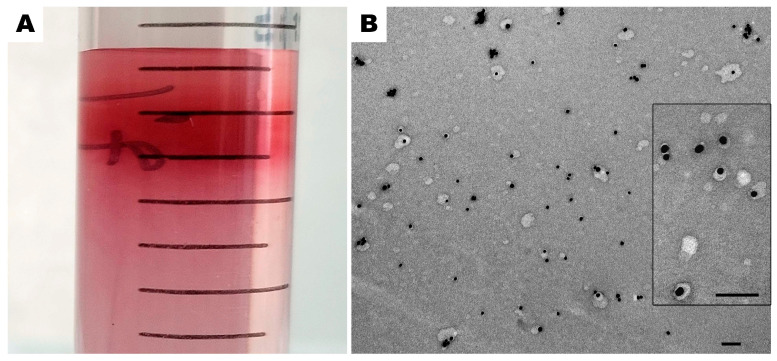
(**A**)—Photo of a test tube with MLNC-2 after centrifugation on a cushion with 7% sucrose, 300× *g* 140 min. (**B**)—representative images (enlarged in insert) of MLNC-2 in colored layer of 7% sucrose. TEM, negative staining with 0.5% uranyl acetate. Length of scale bars corresponds to 100 nm.

To overcome this difficulty, we reduced the centrifugation rate to 300× *g* to preserve the MLNC-2 structure while maintaining separation on a 7% sucrose cushion. This value was chosen because it is safe for centrifuging eukaryotic cells and does not damage the integrity of cellular membrane structures. Because the acceleration was reduced, we increased the centrifugation time from 15 to 140 min. After centrifugation, we observed the stained layer containing MLNC-2 diffusing into the sucrose cushion (Figure 5A).

A brightly colored layer was collected, in which TEM imaging revealed MLNC-2 coated with a lipid envelope (electron transparent material), a small number of empty lipid particles, and electron-dense cores (Figure 5B). According to DLS data, the particles had a hydrodynamic diameter of 36.1 ± 16.3 nm (Figure S1A), and the polydispersity index was 0.282.

The obtained results show that the main challenge in purifying the resulting MLNC-2 suspension is the mechanical strength of the particle lipid envelope. Overall, our previously proposed [26] approach to obtaining MLNC-type particles can be adapted to various lipid compositions, regardless of the charge of the lipids used. However, it should be taken into account that the strength limit of the lipid envelope depends on its composition; therefore, to create a MLNC with a new lipid composition, an individual selection of isolation conditions will be required.

### 3.6. Interaction of MLNC-2 with Human Albumin

**Figure 6:**
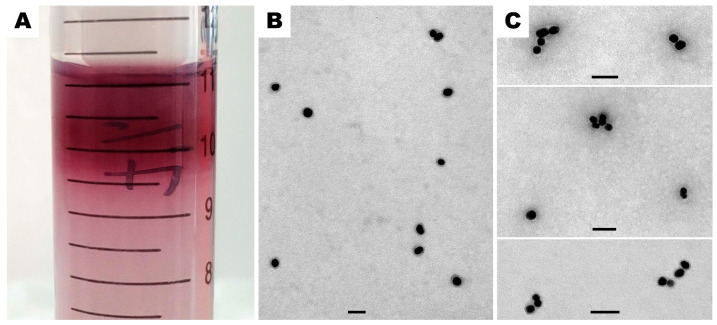
(**A**)—Photo of a test tube with MLNC-2/HSA after centrifugation on a cushion with 7% sucrose, 300× *g* 140 min. (**B**)—Representative TEM images of MLNC-2/HSA in colored layer of 7% sucrose. (**C**)—Representative images of cores incubated with HSA and centrifuged on a cushion with 7% sucrose, 300× *g* 140 min. Thin homogeneous layer of middle electron density is hardly visible around the cores both in Figures B and in C. Negative staining with 0.5% uranyl acetate. Length of scale bars corresponds to 50 nm.

We were interested in how the presence of a protein corona on the surface of MLNC-2 would affect the strength of the lipid envelope. To know this, we incubated MLNC-2 particles with an HSA solution (4.059 mg/mL) for 15 min at room temperature, followed by centrifugation under optimized conditions. It should be noted that after centrifugation, the MLNC-2/HSA sample has a more purple color (Figure 6A) than the original MLNC-2 preparation (Figure 5A), indicating cores adhesion.

The most colored fraction was collected, which, according to DLS data, contained three groups of particles with sizes of 53.9 ± 25.8 nm (presumably MLNC-2 with a corona); 3.8 ± 1.0 nm and 1.4 ± 0.2 nm (corresponding to the forms of HSA in solution) (Figure S2A). However, the TEM analysis did not recognize the lipid envelope on surfaces of MLNC-2 particles (Figure 6B). A hardly visible thin layer of middle electron density material probably represents “leftovers” of a layer decorated with HSA.

We assumed that interaction of HSA with the MLNC-2 surface weakened the lipid envelope causing destabilization of MLNC-2. Centrifugation in a viscous medium resulted in the release of the cores and the formation of a protein corona directly on the surface of the AuNPs. To confirm this hypothesis, we prepared cores with a HSA hard corona and centrifuged them. Particles in the collected fraction had a size of 53.5 ± 27.9 nm (Figure S2B), which corresponded to the size parameters of particles isolated from the MLNC-2/HSA sample. TEM analysis also revealed complete similarity between cores with an HSA corona and particles isolated from the MLNC-2/HSA sample (Figure 6C). This data suggests that the formation of a protein corona on the surface of MLNC-type particles may lead to a decrease in the strength of the lipid envelope, which may subsequently hinder the isolation of corona-bearing MLNC particles

### 3.7. MLNC-2 Interaction with Fetal Bovine Serum

Incubation of MLNC-2 with a HSA protein solution resulted in the formation of a protein corona consisting exclusively of this protein, with damage to the MLNC-2 lipid envelope detected. The results obtained indicate that the MLNC-2 lipid envelope loses its integrity in the presence of serum albumin, resulting in the release of cores during MLNC-2 purification. Accordingly, the protein corona will form on the surface of the cores, not the lipid envelope. This raises the question: how will incubation of a protein mixture with MLNC-2 affect the lipid envelope? Since albumin is the major serum protein, a similar effect can be expected when incubating MLNC-2 in a serum solution. To answer this question, we compared the protein composition of the hard corona on MLNC-2 (1) and on the newly synthesized cores (2) after purification. The compositions of the hard coronas should differ significantly if, upon incubation with serum, the lipid envelope of MLNC-2 remains intact and the corona is formed on the lipid envelope. In addition, we prepared and examined photo-fixed full corona on the lipid surface of MNLC-2 for control of differences with hard corona. MLNC-2 particles bearing the protein corona were prepared using 10% fetal bovine serum (FBS) and incubated with native FBS or FBS modified with a photoactivated cross-linker PACL.

**Figure 7:**
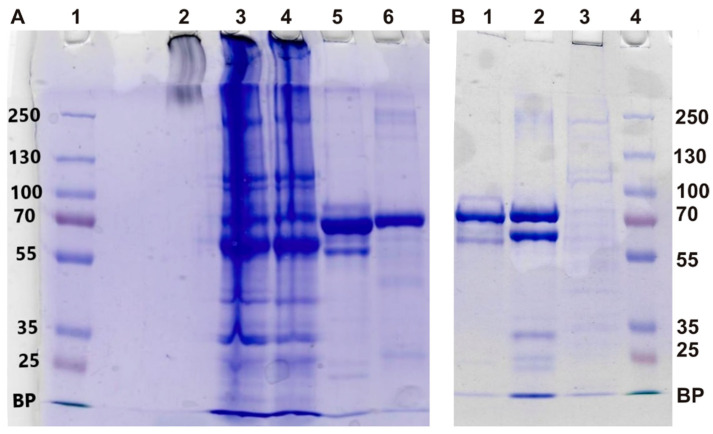
Scanned image of Coomassie-stained polyacrylamide gel. (**A**): 1—Protein mass marker, masses are indicated on the left, kDa; 2—MLNC-2; 3—MLNC-2/HC; 4—MLNC-2/PFC; 5—FBS; 6—bovine serum albumin (BSA). (**B**): 1—FBS; 2—BSA; 3—core/HC; 4—protein mass marker. BP—bromophenol.

Samples incubated with PACL-modified FBS, similar to [22], were subjected to UV photofixation to obtain MLNC-2/PFC (particles bearing a full photo-fixed corona). In the case of using non-modified FBS, MLNC-2/HC (hard corona particles) were obtained. Both types bearing protein corona particles were isolated by centrifugation on a 7% sucrose cushion. The resulting particles were washed three times with a 7% sucrose solution to remove unbound serum proteins. For comparison, newly prepared cores and cores purified on a sucrose cushion with a hard corona consisting of FBS proteins were prepared. The presence of protein in the MLNC-2/HC and MLNC-2/PFC and core/HC samples was determined by Laemmli gel electrophoresis (Figure 7).

The electropherogram profiles for MLNC-2/HC and MLNC-2/PFC are similar, indicating a similar composition of the coronas. The major protein of FBS is albumin (69 kDa); however, the major protein of the MLNC-2 protein corona has a smaller mass, about 60 kDa. Also, as in the case of previously studied MLNC-1 [22,32], the MLNC-2 corona contains minor serum protein components (Figure 7A, lanes 3, 4), which are not visualized in FBS (Figure 7A, lane 5).

**Figure 8:**
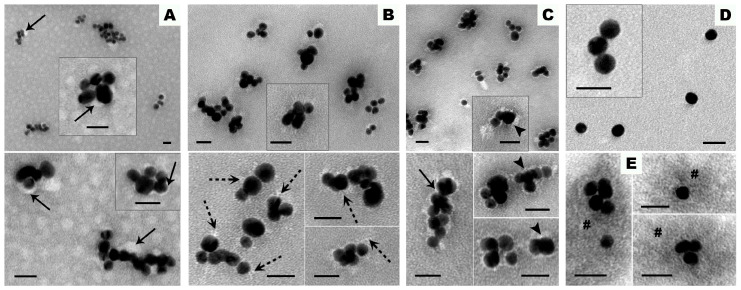
Representative images of MLNC-2 and core variants at different magnifications. Column (**A**)—purified MLNC-2, Column (**B**)—MLNC-2/HC, Column (**C**)—MLNC-2/PFC, (**D**)—newly synthesized cores, (**E**)—core/HC. The arrow shows the lipid envelope; the dashed arrow—the hard corona; the arrowhead—the photo-fixed full corona, #—FBS components. TEM, negative staining with 0.5% of uranyl acetate. The length of scale bars corresponds to 25 nm.

When comparing the electropherograms of MLNC-2 bearing a hard or photo-fixed full corona (Figure 7A, lanes 3 and 4) with those of cores (Figure 7B, lane 3), a clear difference in the intensity of protein material staining is evident. This indicates that in lanes 3 and 4 (Figure 7A), significantly more serum protein is bound to MLNC-2 than to the same amount of core/HC (Figure 7B, lane 3). Electropherograms clearly show that MLNC-2/HC binds a greater amount of proteins from FBS (Figure 7A, lane 3) than the same amount of core/HC (Figure 7B, lane 3). This indicates that the MLNC-2 lipid envelope is preserved in the serum solution during the isolation process. To confirm the preservation of the MLNC-2 structure and the formation of a protein corona on its lipid surface, these samples were examined by TEM (Figure 8).

The lipid envelope on the MLNC-2 is visualized as an electron-transparent material with a smooth border (Figure 8A), while electron-transparent “shreds” and short filaments are visible on the MLNC-2/HC surface (Figure 8B). On the core/HC surface (Figure 8E), an electron-transparent material is not visible, consistent with the absence of a lipid membrane. The core/HC surface is similar to the surface of newly synthesized cores (Figure 8D). It is worth noting that we were previously unable to visualize signs of a hard corona on MLNC-1 in TEM [32], whereas for MLNC-2 we observe changes in the surface structure of the particles.

Photofixation of the full protein corona on the surface of MLNC-2 allows visualization of a continuous electron-transparent layer of the lipid envelope around all particles of MLNC-2/PFC. The surface of full protein corona demonstrated the “teeth”, protrusions and small clusters of electron-transparent loose material of various shapes are visible (Figure 8C) and are more numerous and better defined than in the MLNC-2/HC sample.

The obtained results demonstrate that the properties of the MLNC-2 lipid envelope (PC/PA/Chol (7:2:1)) vary depending on the composition of the incubation medium. Thus, when albumin (HSA) is adsorbed onto the MLNC-2 lipid surface, the lipid membrane destabilizes, as described in Section 3.6. MLNC-2/HC particles fail to withstand a centrifugation load of 300× *g*, the lipid membrane is disrupted, and the cores are released into the environment. Incubation of MLNC-2 in 10% FBS results in the formation of a hard corona and preserves the lipid membrane, allowing for the isolation of MLNC-2/HC.

It can be supposed that albumin undergoes conformational changes and actively interacts with envelope lipids [33], leading to its destabilization. Apparently, other serum proteins, present in FBS, adsorb to the lipid surface more rapidly and interact with it differently than albumin, thereby shielding the surface from it. Thus, incubation in serum maintains the integrity of the PC/PA/Chol (7:2:1) lipid envelope, allowing the isolation of MLNC-2/HC and MLNC-2/PFC particles.

## 4. Conclusions

We demonstrated that our previously developed approach to the assembly and purification of MLNC-1-type particles [26] could be modified to obtain particles with a similar structure that carry a negatively charged lipid coating. We obtained a multilevel nanoconstruct using a negatively charged core and a negatively charged lipid envelope. Magnesium ions, at a low concentration, served as the “glue” between the two negatively charged components. Thus, we reproduced a previously developed approach, demonstrating its flexibility, and obtained the desired nanoparticles that are stable and easy to manipulate.

An adapted purification protocol for novel MLNC-2 can be used to isolate MLNC-2 particles bearing a hard protein corona or a photo-fixed full corona on their surface. However, the success of this procedure depends on the interaction between the corona proteins and the lipid envelope. A corona composed of albumin leads to destabilization of the lipid envelope by the PC/PA/Chol (7:2:1) composition, which disappears during purification. A corona formed by serum proteins does not disrupt the lipid envelope, allowing the isolation of MLNC-2 particles bearing a protein corona on their lipid surface. This phenomenon possibly reflects a specific interaction between albumin and the lipid membrane of the PC/PA/Chol (7:2:1) composition.

Summarizing the results of this work and our previous studies [24,26], we can conclude that the MLNC-type particles we proposed can successfully serve as a universal platform for studying the protein corona formed on the lipid surface.

## Figures and Tables

**Table 1:** Examination of particle ζ-potential as a function of the proportion of PA in the lipid mixture.

Lipid Composition PC/PA/Cholesterol, mol%	ζ-Potential ± SD, mV
80/10/10	−12.0 ± 4.9
70/20/10	−37.8 ± 7.2
50/40/10	−33.2 ± 9.5
40/50/10	−39.4 ± 8.9

## Data Availability

The original contributions presented in this study are included in the article/Supplementary Material. Further inquiries can be directed to the corresponding author.

## Supplementary Materials

The following supporting information can be downloaded at: https://www.mdpi.com/article/10.3390/nano16120743/s1, Figure S1: title Characterization of MLNC-2, purified by centrifugation on a 7% sucrose cushion, by DLS for three independent preparations: A – d = 36 ± 16 nm, PdI = 0.282; B – d = 32 ± 14 nm, PdI = 0.266; C – d = 35 ± 17 nm, PdI = 0.312. Figure S2: title Characterization of MLNC-2/HSA (A, d = 54 ± 26 nm, PdI=0.323) and core/HSA (B, dH = 53 ± 28 nm, PdI = 0.414), purified by centrifugation on a 7% sucrose cushion, by DLS.

## References

[1] Winkeljann B., Lapuhs P., Alonso M.J., Kimna C. (2026). A multiscale approach to lipid nanoparticle engineering from molecular structure to in vivo performance. J. Control. Release.

[2] Wang R., Wang H., Li T., Zi G., Yang Z., Xu Y., Peng B. (2026). A rapid and reliable strategy for identification of LNP and protein corona in vitro and in vivo. Int. J. Pharm. X.

[3] Xu W., Zhu S., Sun Z., Ye J., Chu H. (2026). Self-assembled nanoplatforms for cancer immunotherapy: Principles, progress and perspectives. Acta Pharm. Sin. B.

[4] Shadab A., Haider M.F., Abohassan M. (2026). Liposome-Based Drug Delivery Systems: Mechanisms, Preparation Strategies, Clinical Status, and Therapeutic Applications. AAPS PharmSciTech.

[5] Mehta M., Bui T.A., Yang X., Aksoy Y., Goldys E.M., Deng W. (2023). Lipid-Based Nanoparticles for Drug/Gene Delivery: An Overview of the Production Techniques and Difficulties Encountered in Their Industrial Development. ACS Mater. Au.

[6] Chu R., Wang Y., Kong J., Pan T., Yang Y., He J. (2024). Lipid Nanoparticles as the Drug Carrier for Targeted Therapy of Hepatic Disorders. J. Mater. Chem. B.

[7] Arabestani M.R., Bigham A., Kamarehei F., Dini M., Gorjikhah F., Shariati A., Hosseini S.M. (2024). Solid Lipid Nanoparticles and Their Application in the Treatment of Bacterial Infectious Diseases. Biomed. Pharmacother..

[8] Zhang C., Ma Y., Zhang J., Kuo J.C.-T., Zhang Z., Xie H., Zhu J., Liu T. (2022). Modification of Lipid-Based Nanoparticles: An Efficient Delivery System for Nucleic Acid-Based Immunotherapy. Molecules.

[9] Li Y., Ye Z., Yang H., Xu Q. (2022). Tailoring Combinatorial Lipid Nanoparticles for Intracellular Delivery of Nucleic Acids, Proteins, and Drugs. Acta Pharm. Sin. B.

[10] Charbgoo F., Nejabat M., Abnous K., Soltani F., Taghdisi S.M., Alibolandi M., Thomas Shier W., Steele T.W.J., Ramezani M. (2018). Gold nanoparticle should understand protein corona for being a clinical nanomaterial. J. Control. Release.

[11] Zhao Y., Liu C., Zhang X., Gu T., Wang Y., Zuo T., Liu S., Xu X., Meng H. (2026). Mechanistic and Strategic Insights into How Lipid Nanoparticles Interact with Membrane-Based Biological Barriers. ACS Nano.

[12] Canchola A., Li K., Chen K., Ukbamichael A., Wang X., Chou W.C. (2026). Advancing nanomedicine with machine learning: Predicting protein corona and nano-bio interactions. Nanomedicine.

[13] Abo Qoura L., Kostyushev D., Parodi A., Boyarintsev D.I., Chulanov V., Pokrovsky V.S. (2026). Nanoparticle-Host Interactions: The Impact of Physiological and Pathological Factors on Biodistribution, Immune Processes, and Translational Challenges. Cell. Mol. Bioeng..

[14] Navid Talemi M., Ramezani Farani M., Alipour Eskandani N., Mirzaee D., Alipourfard I., Huh Y.S. (2026). Programmable lipid nanoparticles for RNA therapeutics: Design principles and clinical translation. Mater. Today Bio.

[15] García-Álvarez R., Vallet-Regí M. (2021). Hard and Soft Protein Corona of Nanomaterials: Analysis and Relevance. Nanomaterials.

[16] Lee Y.K., Choi E.J., Webster T.J., Kim S.H., Khang D. (2014). Effect of the protein corona on nanoparticles for modulating cytotoxicity and immunotoxicity. Int. J. Nanomed..

[17] Liu N., Tang M., Ding J. (2020). The interaction between nanoparticles-protein corona complex and cells and its toxic effect on cells. Chemosphere.

[18] Fleischer C.C., Payne C.K. (2014). Nanoparticle-cell interactions: Molecular structure of the protein corona and cellular outcomes. Acc. Chem. Res..

[19] Gatti L., Chirizzi C., Rotta G., Milesi P., Sancho-Albero M., Sebastián V., Mondino A., Santamaría J., Metrangolo P., Chaabane L. (2023). Pivotal role of the protein corona in the cell uptake of fluorinated nanoparticles with increased sensitivity for 19F-MR imaging. Nanoscale Adv..

[20] Mohammad-Beigi H., Hayashi Y., Zeuthen C.M., Eskandari H., Scavenius C., Juul-Madsen K., Vorup-Jensen T., Enghild J.J., Sutherland D.S. (2020). Mapping and identification of soft corona proteins at nanoparticles and their impact on cellular association. Nat. Commun..

[21] Morbidelli M., Papini E., Tavano R. (2024). Essential protocols for decoding the composition and the functional effects of the nanoparticle protein corona. Front. Nanotechnol..

[22] Epanchintseva A.V., Baranova S.V., Poletaeva J.E., Tupitsyna A.V., Ryabchikova E.I., Dovydenko I.S. (2024). The Photomodification Method Allows for Determining the Composition of the Full and Soft Protein Corona on the Lipid Surface of Composite Nanoparticles. Nanomaterials.

[23] Poletaeva J., Dovydenko I., Epanchintseva A., Korchagina K., Pyshnyi D., Apartsin E., Ryabchikova E., Pyshnaya I. (2018). Non-Covalent Associates of siRNAs and AuNPs Enveloped with Lipid Layer and Doped with Amphiphilic Peptide for Efficient siRNA Delivery. Int. J. Mol. Sci..

[24] Epanchintseva A.V., Poletaeva J.E., Bakhno I.A., Belov V.V., Grigor’eva A.E., Baranova S.V., Ryabchikova E.I., Dovydenko I.S. (2023). Fixation and Visualization of Full Protein Corona on Lipid Surface of Composite Nanoconstruction. Nanomaterials.

[25] Poletaeva J.E., Chelobanov B.P., Epanchintseva A.V., Tupitsyna A.V., Dovydenko I.S., Ryabchikova E.I. (2025). Internalization of Lipid-Coated Gold Nanocomposites and Gold Nanoparticles by Mouse SC-1 Fibroblasts in Monolayer and Spheroids. Nanomaterials.

[26] Epanchintseva A.V., Poletaeva J.E., Dovydenko I.S., Chelobanov B.P., Pyshnyi D.V., Ryabchikova E.I., Pyshnaya I.A. (2021). A Lipid-Coated Nanoconstruct Composed of Gold Nanoparticles Noncovalently Coated with Small Interfering RNA: Preparation, Purification and Characterization. Nanomaterials.

[27] Ellington A., Pollard J.D. (1998). Synthesis and Purification of Oligonucleotides. Curr. Protoc. Mol. Biol..

[28] Frens G. (1973). Controlled Nucleation for the Regulation of the Particle Size in Monodisperse Gold Suspensions. Nat. Phys. Sci..

[29] Liu X., Atwater M., Wang J., Huo Q. (2007). Extinction coefficient of gold nanoparticles with different sizes and different capping ligands. Colloids Surf. B Biointerfaces.

[30] Amaya-González S., López-López L., Miranda-Castro R., de-los-Santos-Álvarez N., Miranda-Ordieres A.J., Lobo-Castañón M.J. (2015). Affinity of Aptamers Binding 33-Mer Gliadin Peptide and Gluten Proteins: Influence of Immobilization and Labeling Tags. Anal. Chim. Acta.

[31] Epanchintseva A.V., Gorbunova E.A., Ryabchikova E.I., Pyshnaya I.A., Pyshnyi D.V. (2021). Effect of Fluorescent Labels on DNA Affinity for Gold Nanoparticles. Nanomaterials.

[32] Epanchintseva A.V., Baranova S.V., Poletaeva J.E., Bakhno I.A., Ryabchikova E.I., Dovydenko I.S. (2024). Study of Hard Protein Corona on Lipid Surface of Composite Nanoconstruction. Nanomaterials.

[33] Park S.J. (2020). Protein-Nanoparticle Interaction: Corona Formation and Conformational Changes in Proteins on Nanoparticles. Int. J. Nanomed..

