# Supplementary material for: Assembly of Multilevel Nanoconstructs with Negatively Charged Lipid Envelope and Features of Its Interaction with Protein Corona
Source: Nanomaterials (Basel). 2026 Jun 14;16(12):743. doi: 10.3390/nano16120743 (PMC13305301; doi:10.3390/nano16120743)
Supplement: Supplementary file 1 [file nanomaterials-16-00743-s001.zip › nanomaterials-4321906-supplementary.pdf]

Supporting information for article

“Assembly of multilevel nanoconstructs with negatively charged lipid envelope and features of its interaction with protein corona”

Ilya S. Dovydenko[1,†], Anna V. Epanchintseva[1,†], Julia E. Poletaeva[1] and Elena I. Ryabchikova[1,*]

Institute of Chemical Biology and Fundamental Medicine, Siberian Branch of Russian Academy of Science, Lavrent’ev avenue. 8, 630090 Novosibirsk, Russia

† These authors contributed equally to this work.

* Correspondence: lenryab@niboch.nsc.ru.

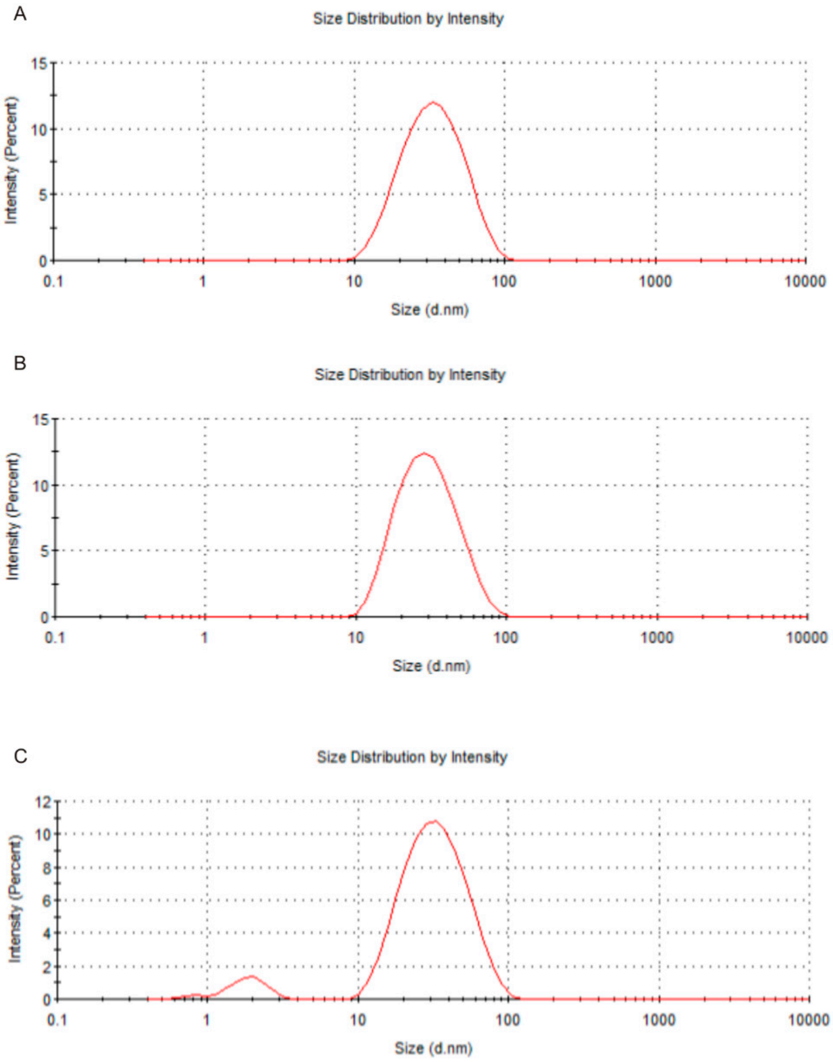

Figure S1. Characterization of MLNC2, purified by centrifugation on a 7% sucrose cushion, by DLS for three independent preparations: A – d = 36 ± 16 nm, PdI = 0.282; B – d = 32 ± 14 nm, PdI = 0.266; C – d = 35 ± 17 nm, PdI = 0.312.

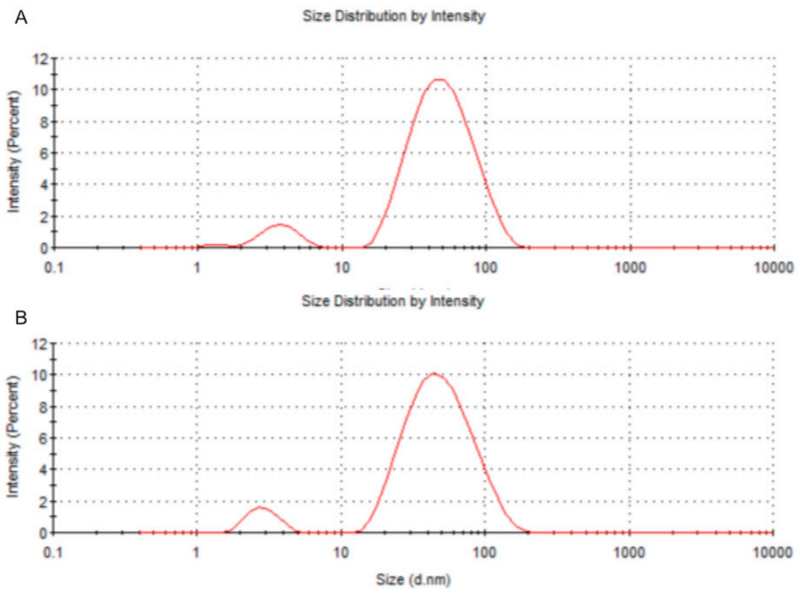

Figure S2. Characterization of MLNC2/HAS (A, d=54 ± 26 nm, PdI=0.323) and core/HSA (B, dH=53 ± 28 nm, PdI=0.414), purified by centrifugation on a 7% sucrose cushion, by DLS.
